# Identification and validation of reference genes for RT-qPCR analysis in *Iris domestica* under Cd stress

**DOI:** 10.1016/j.heliyon.2024.e36923

**Published:** 2024-08-24

**Authors:** Beibei Su, Ziwei Li, Hongli Liu, Xiaoyun Niu, Xiaojie Zhao, Yumeng Wu, Qian Wang, Yangchen Yuan, Zhuolin Xiao, Dazhuang Huang

**Affiliations:** a College of Landscape Architecture and Tourism, Hebei Agricultural University, Baoding, 071000, China; b Shijiazhuang Information Engineering Vocational College, Shijiazhuang, 052161, China; c College of Landscape Architecture and Arts, Henan Agricultural University, Zhengzhou, 450002, China

**Keywords:** *Iris domestica*, RT-qPCR, Cd stress, Reference genes

## Abstract

*Iris domestica* is a widely used ornamental garden and important medicinal plant. Our previous studies have shown that it exhibits significant uptake and translocation capacity under Cd stress compared to other *Iris* species. Gene expression is studied using RT-qPCR; however, there are no reference genes have been found for *I. domestica* under Cd stress. In this investigation, thirteen possible reference genes from previous studies and our transcriptome were screened using RT-qPCR in the leaves and roots of Cd-stressed plants. The findings revealed that *UBC9* and *ACT* were the best reference genes for roots with and without Cd stress, whereas *YLS8* and *ACT7* were the best reference genes for leaves. Among the different tissues without Cd stress, *UBC9* and *UBC28* exhibited the best results, whereas *PP2C06* and *UBC9* exhibited the best results under Cd stress. The most stable reference genes in the leaves and roots were *UBC9* and *UBC28*, respectively, under and without Cd stress, and *GADPH* was the most unstable. Finally, three metal ion response genes, *NRAMP2*, *YSL9* and *CYP81Q32* were detected using RT-qPCR and compared with the transcriptome data to further confirm the reliability of the chosen genes. This study identified suitable reference genes for *I. domestica* under Cd-stress conditions.

## 1 Introduction

*Iris domestica* is a perennial herb belonging to the Iridaceae family, and is an important medicinal and ornamental plant. The flowering period occurs throughout the hot summer. Because the male parent material of the distant hybridization of *I.* × *norrisii* is rich in color, it has been favored by gardening artists in recent years [1,2]. *I. domestica* is a traditional medicinal plant with rhizomes and flavonoids as its main active components [3]. To date, most of the research on *I*. *domestica* is about its medicinal value [[3], [4], [5]] and on distant crossbreeding [6,7]. Soil heavy metal (HM) pollution has been becoming serious and widespread issue induced by industrial and agricultural activities, which has detrimental influences on plant growth, water purification, and food safety [8]. Lead, arsenic, cadmium, chromium, and mercury are the most frequent heavy metals that cause poisonings in humans [8]. It is quite important to examine the reaction molecular mechanism of plants to cadmium and explore ornamental plants that can be used for remediation of polluted environment. *Iris* plants are often cultivated for landscaping in harsh environments owing to their excellent resistance to drought, salt, and heavy metals [[9], [10], [11], [12], [13], [14], [15], [16]]. By comparing the responses of *I. domestica* with those of other *Iris* species to the heavy metals Pb and Cd, we found that most *Iris* species store heavy metals in their roots to protect their aboveground parts from harm. However, *I. domestica* has a good ability to absorb and transport heavy metals, especially Cd, and its content in the aboveground parts is significantly higher than that in the other areas. The ecological restoration of soils contaminated by garden plants warrants further research. Owing to a lack of genomic information, molecular biology research on *I. domestica* is relatively limited. Transcriptome sequencing and gene expression analysis are necessary to elucidate several the underlying molecular mechanisms.

Quantitative reverse transcription polymerase chain reaction (RT-qPCR) is a molecular biological technique that measures each cycle of amplification produces fluorescence and amplification during the exponential phase in order to quantify templates [17,18]. However, RT-qPCR data can be influenced by various factors such as RNA extraction, reverse transcription [19]. The ideal reference gene should be stably expressed at different developmental stages and physiological conditions [20]. Reference genes in plants include glyceraldehyde-3-phosphate dehydrogenase (*GAPDH*), 18S rRNA (*18S*), actin (*ACT*), and β-tubulin (*β-TUB*), are usually involved in maintaining the basic metabolic activities of cells, and their metabolic expression is moderate and stable [21]. However, some of these methods deviate from different plant locations and growth stages, leading to inaccurate RT-qPCR results. In addition, with the wide application of transcriptomics, some new genes, which can also be used as reference genes and have better stability than other genes, exhibit stable expression under specific conditions [22,23]. For example, based on transcriptomic data, some new genes in *Lindera megaphylla* have been screened for use as reference genes and fully validated under various stresses and in different materials [24]. Based on transcriptome data, traditional and commonly used reference genes have been accurately compared during cherry radish root expansion [25]. Scholars have performed a series of studies on the reference genes of the genus *Iris*. For instance, in *I. germanica* L., *ACT11* and elongation factor 1α (*EF1α*) found to be the genes that varied the least across different tissues [26], while ubiquitin-protein ligase (*UBC*) and *GAPDH* were found to be the most stable reference genes for different phases of flower development [27]. Research on *I. domestica* revealed that *EF1β* was the most appropriate gene in different tissues under drought stress [28]. For *I. lactea* var. *chinensis* roots exposed to lead, salt, and Cd stress, eukaryotic initiation factor 5A (*EIF-5A*), histones (*HIS*), and *UBC* are the best reference genes [29]. In conjunction with the transcriptome data obtained from *I. domestica* under Cd stress, we discovered that certain genes exhibited low and unstable expression levels, while other genes had more stable expression. In conjunction with the transcriptome data obtained from *I. domestica* under Cd stress, we that discovered. As a result, choosing the right reference genes is crucial to guaranteeing the correctness of RT-qPCR results.

In this study, we conducted preliminary screening of the reported reference genes of *I. domestica*. We selected three stable reference genes, *UBC9*, *GADPH*, and *ACT*, as candidates, and used previously the primers listed [3,16,28]. Second, from the transcriptome, 10 reference genes, including *ACT7*, *UBC28*, splicing Factor U2af large subunit B-like (*U2AF65B*), TATA-box binding protein like 1 (*TBP1*), thioredoxin-like protein YLS8 (*YLS8*), eukaryotic initiation factor 4A-III homologue A-like (*EIF4A3A*), ribosomal protein L8 (*RPL8*), protein phosphatase 2A (*PP2A1*), tubulin β-1 chain (*TUBB1*), and protein phosphatase 2C 6 (*PP2C06*), were screened based on the following criteria: values ranging from 30 to 180 to 1.5-fold for transcript fragments per lilobase of exon model per million mapped fragments (FPKM). RT-qPCR was used to assess the stability of the 13 potential reference genes in roots and leaves of *I. domestica* during Cd stress. Statistical software applications such as Delta cycle threshold (ΔCt) [13], geNorm [30], NormFinder [31] and BestKeeper [32] are statistical software programs used to evaluate the stability of candidate reference genes from various perspectives. RefFinder is an online analysis software that combines ΔCt, geNorm, BestKeeper, and NormFinder [33]. NRAMP and YSL belong to the natural resistance-associated macrophage protein (NRAMP) family of metal transporter proteins and the yellow stripe-like (YSL) protein family. Several studies have shown that they play crucial roles in the uptake and transport of heavy metals [[34], [35], [36]]. Isoflavones are the main medicinal ingredients of *I. domestica* [3], and the expression of isoflavone 3′-hydroxylase-like (*CYP81Q32*) is significantly different in the presence of Cd. Our transcriptome data showed that *NRAMP2*, *YSL9*, and *CYP81Q32* were differentially expressed, with relatively high FPKM values. Thus, we assessed the expression of *NRAMP2*, *YSL9*, and *CYP81Q32* using stable and unstable genes and compared the results with the FPKM values of the transcriptomes of these genes to bolster the validity of the previously discussed findings.

## 2 Materials and methods

### 2.1 Plant materials and Cd treatment

*I. domestica* seeds were obtained from Anguo, Baoding City (38°15′ N, 115°10′ E). On November 1, 2021, seeds were sown on a nutrient substrate and cultured in an intelligent greenhouse at Hebei Agricultural University, Hebei Province*.* Uniform plants with heights of approximately 15 cm were selected in April 2022. The roots were washed and pre-incubated in a 1/4 Hoagland Nutrient Solution for 15 days. Subsequently, the plants were transferred to a 1/2-strength Hoagland medium supplemented with varying concentrations of CdCl_2_ (0, 5, or 50 mg/L, i.e., Cd0, Cd5, or Cd50) for seven days. During the incubation period, the plants were continuously aerated and maintained at a constant water level and pH of 6.5. At the conclusion of the trial, foliage and underground parts were gathered from the identical site and promptly preserved in liquid nitrogen. In order to prepare them for transcriptome sequencing and RNA extraction, the samples were kept at −80 °C. Every sample was examined in three biological duplicates. The experimental subgroups are listed in Table 1.

**Table 1:** Experimental grouping details.

Group Sets	Sample Types	Description
Total	Cd0-L; Cd5-L; Cd50-L Cd0-R; Cd5-R; Cd50-R	No stress, under Cd stress, all samples of roots and leaves of seedlings.
Tissues	Cd0-L; Cd0-R	The roots and leaves of unstressed seedlings.
Stress	Cd5-L; Cd50-L Cd5-R; Cd50-R	The roots and leaves of seedlings under different concentrations of Cd stress.
Leaves	Cd0-L; Cd5-L; Cd50-L	Leaves of seedlings under no stress and Cd stress.
Roots	Cd0-R; Cd5-R; Cd50-R	Roots of seedlings under no stress and Cd stress.

### 2.2 Extraction of total RNA and synthesis of cDNA

Total RNA was isolated using an RNA extraction kit from HUAYUEYANG Biotechnology. Using a 1.5 % (w/v) agarose gel electrophoresis and a Nanodrop 2000 spectrophotometer, the RNA's integrity and concentration was assessed. The Evo M-MLV RT Mix Kit and gDNA Clean for qPCR Ver.2 (Accurate Biotechnology, Hunan, China) were used to synthesize first-strand cDNA, as directed by the manufacturer. For 2 min, a combination containing 1 μg of total RNA, 2 μL of gDNA Clean Reaction Mix Ver.2, and 10 μL of RNase-free water devoid of RNase were incubated at 42 °C. Then, 10 μL of the cleaned RNA, 4 μL of 5 × Evo M-MLV RT Reaction Mix Ver.2, and 6 μL of RNase-free water were added to start the cDNA synthesis process. These were then incubated at 37 °C for 15 min and 85 °C for 5 s. In order to use the resultant cDNA in later RT-qPCR experiments, it was diluted five times.

### 2.3 Selection and design of primers for candidate reference genes

Three reported reference genes in *I. domestica*, *GADPH*, *UBC9*, and *ACT,* were selected and the primers used are listed in the text. Finally, ten candidate genes (*ACT7*, *U2AF65B*, *TBP1*, *YLS8*, *EIF4A3A*, *RPL8*, *PP2A1*, *UBC28*, *TUBB1,* and *PP2C06*) were selected from the transcriptome database and reference genes reported in literature for *I. domestica.* The design of the primers involved the utilization of the Primer Quest Tool (http://sg.idtdna.com/Primerquest/Home/Index) with reference to coding sequences obtained from the transcriptome and data sourced from the NCBI database (https://www.ncbi.nlm.nih.gov/). Priority was given to selecting primers in close proximity to the 3′- end of the gene. Comprehensive details regarding the 13 candidate primers employed for reference genes are provided in Table 2.

**Table 2:** Design of RT-qPCR primers for candidate reference genes.

Gene name	Sequence (5′-3′)	Standard Curve	R^2^	E (%)	Product Length(bp)
*UBC9*	F: AGGAGGTTCGTGCGTGTT	y = −3.3802x + 9.7476	0.9978	97.624	149
	R: GGGGACCCATTATTGTTGC				
*GADPH*	F: GGCGATCTCTTTCTCTATCCC	y = −3.4262 x + 9.4300	0.9980	95.825	179
	R: GCTGGATGCTTCTACCACT				
*Actin*	F: TGCCATGTATGTCGCTATCCAG	y = −3.141x + 8.9924	0.9958	108.147	122
	R: GTGCATATCCTTCATAAATTGGAAC				
*YLS8*	F: CTTGGAACCGGAAACAACAAC	y = −3.1168x + 9.7143	0.9956	109.335	112
	R: TGACAAGACCACGACCTTTC				
*RPL8*	F: GGCAATGCTTACCACAAGTTC	y = −3.5385x + 10.267	0.9958	91.693	82
	R: GATGCTCCACAGGGTTCATAG				
*UBC28*	F: CCAGAAGGAGCTTCAGGATTT	y = −3.2157x + 13.035	0.9964	104.632	122
	R: CAGAATAAGGACTGTCCGAAGG				
*TUBB1*	F: CTTGTTGAGAACGCTGATGAATG	y = −3.378x + 10.396	0.9991	97.712	120
	R: GGCAGAGATAAGGTGGTTTAGG				
*PP2C06*	F: GCACTGTCGGTGGATCATAAA	y = −3.0022x + 11.251	0.9933	115.322	101
	R: CCGAACACACGATATCCATTCC				
*U2AF65B*	F: GGGTGGTCTTCCATACTACTTTAC	y = −2.7548x + 10.807	0.9914	130.676	99
	R: GTTTCCCTGTCCTTCACAAGA				
*ACT7*	F: TCTTACTGAAGCGCCTCTAAAC	y = −3.3305x + 8.5017	0.9999	99.644	128
	R: GTCCACTGGCATATAGGGAAAG				
*TBP1*	F: GCTAGAGGGAGAAGGTTCTTTG	y = −3.1227x + 11.263	0.9982	109.043	103
	R: CAACTGCCTCTCAGTGTTATCT				
*EIF4A3A*	F: GTTGACAGAGAAGATGCGTAGT	y = −3.2956x + 10.089	0.9942	101.111	101
	R: CTGACCTGAACTCTGCCATTAT				
*PP2A1*	F: GTCCGATGTGCGATCTTCTT	y = −2.8458x + 12.863	0.9897	124.592	93
	R: CGTCCTGACCGAATGTGTATC				

### 2.4 Detection of the specificity of candidate reference genes

We performed PCR amplification using cDNA templates from six distinct samples to verify the specificity of the proposed primers for the putative reference genes. Ten microliters of 2 × Taq Master Mix, 1 μL each of forward and reverse primers, 1 μL each of cDNA template, and 7 μL of ddH_2_O made up the reaction mixture. Three minutes of initial denaturation at 94 °C, thirty cycles of denaturation at 94 °C for 30 s, annealing at 60 °C for 30 s, and extension at 72 °C for 10 s, and finally 5 min of final extension at 72 °C were the conditions under which the PCR was conducted. Electrophoresis on 1 % (w/v) agarose gel was used to evaluate the PCR results.

### 2.5 Analysis of amplification efficiency and RT-qPCR

To determine the amplification efficiency and curve for each reference gene, cDNA templates from six samples were combined in equal proportions and subjected to a 10-fold serial dilution (10^−1^, 10^−2^, 10^−3^, 10^−4^, and 10^−5^) for RT-qPCR analysis. The standard curve was plotted with Ct values as vertical coordinates and logarithmic values of the dilutions as horizontal coordinates. The correlation coefficient (R^2^), slope (k), and primer amplification efficiency (E) were calculated with the formula: E = (10^*−*1/slope^−1) × 100 %.

RT-qPCR was used the SYBR Green Premix Pro Taq HS qPCR Kit (Accurate Biotechnology, Hunan, China) and a QuantStudioTM5 Real-Time PCR System (Applied Biosystems, Los Angeles, CA, USA). Each 20 μL RT-qPCR reaction mixture contained 10 μL of 2 × SYBR Green Pro Taq HS Premix, 1 μL of cDNA, 0.4 μL of each primer (forward and reverse), 0.4 μL of ROX Reference Dye (4 μM), 7.8 μL of water (RNase-free). The amplification procedure included 40 cycles of 5 s at 95 °C and 30 s at 60 °C after an initial denaturation of 30 s at 95 °C. Following a 30 s initial denaturation at 95 °C, the amplification process comprised 40 cycles of 5 s at 95 °C and 30 s at 60 °C.

### 2.6 Data analysis

The 13 potential reference genes were evaluated for stability using four widely-used algorithms: geNorm [30], NormFinder [31], BestKeeper [32], and the web-based application RefFinder [33] (https://blooge.cn/RefFinder/). With geNorm, Q = 2^−△Ct^ (△Ct = Ct sample – Ct min) is the formula used to convert the Ct value into a more quantitative Q value. The internal reference gene's Ct value for each treatment is represented by Ct sample, while the lowest Ct value for each treatment is shown by Ct min. The expression stability measurement (M) value of each candidate internal reference gene was then calculated using the geNorm tool [37]. The geNorm tool was then used to determine each possible internal reference gene's expression stability M value [37]. Additionally, by computing the pairwise variances in the normalizing factor, geNorm establishes how many reference genes is best. If V_n_/V_n+1_ exceeds 0.15, n+1 is selected. Similar to geNorm, NormFinder bases its ranking of gene stability on the expression stability value (SV), where higher SV values correspond to more stable gene expression [24]. BestKeeper assesses stability by comparing the coefficient of variation (CV) and standard deviation (SD) of the Ct values of candidate genes, with lower CV and SD values indicating greater stability [32]. Specifically, enter the mean value of Ct into the "CP date input" area, and the BestKeeper program will automatically display the calculated correlation coefficient (r), SD and CV in the lower part of the table. Then, according to the decision principle, the more stable internal reference genes were determined. RefFinder (https://blooge.cn/RefFinder/) calculates the geometric mean of the gene expression stability rankings from the three software programs, with a smaller geometric mean indicating greater stability of gene expression [38].

Data processing was performed using Microsoft Excel 2019 software. Line and bar graphs were plotted using the online plotting website Chiplot (https://www.chiplot.online/), while Venn diagrams were plotted using the online plotting software Bioinformatics (https://www.bioinformatics.com.cn/) [39].

### 2.7 Validation of certain reference genes

To ensure the reliability of stable and unstable genes, we analyzed the relative expression of *NRAMP2*, *YSL9* and *CYP81Q32*, using RT-qPCR. These genes were significantly differentially expressed in the sequenced transcriptome of *I. domestica* under Cd stress and had high FPKM values. How accurate the chosen reference genes are reaffirmed through the integration of FPKM values obtained from transcriptome sequencing, providing further validation for their suitability. Using the 2^-ΔΔCt^ technique, the relative gene expression levels were determined. Every experiment was carried out three times.

The primers for *NRAMP2* are (F: 5′-CAGAATTGGGAGGGCTCTTAC-3′ and R: 5′-CGGAACAGTAGGCCATGTATT-3′). The primers for *YSL9* are (F: 5′-GAAGGTGGCTCTCTTCATTCTC-3′ and R: 5′-TGATTAGCCCACACCCTACTA-3′). The primers for *CYP81Q32* are (F: 5′-GGTGGTGAGGTTGAGGAAAT-3′ and R: 5′- GAACTCCGATCATGGTCTTCTC-3′).

## 3 Results

### 3.1 Assessment of the quality of RNA, specificity of primers, and efficiency of amplification

**Fig. 1:**
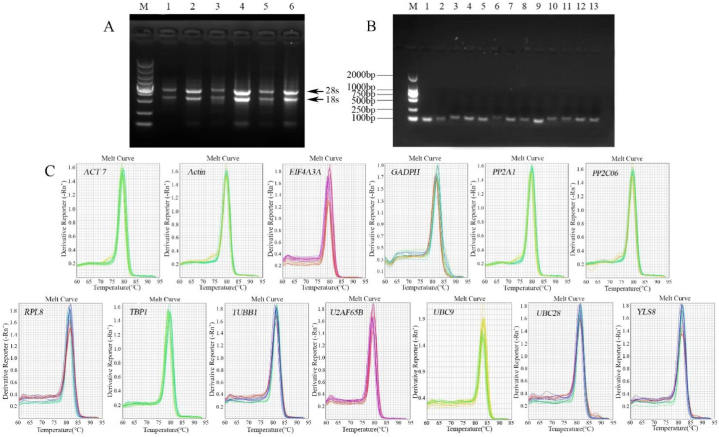
(A) Electrophoresis of RNA extracted from *I. domestica.* Notes: 1: Cd0-L; 2: Cd0-R; 3: Cd5-L; 4: Cd5-R; 5: Cd50-L; and 6: Cd50-R. (B) Electrophoretic analysis of conventional PCR products for 13 candidate genes. Notes: 1: *UBC9* gene; 2: *ACT7* gene; 3: *TBP1* gene; 4: *EIF4A3A* gene; 5: *PP2A1* gene; 6: *GADPH* gene; 7: *Actin* gene; 8: *YLS8* gene; 9: *RPL8* gene; 10: *UBC28* gene; 11: *TUBB1* gene; 12: *PP2C06* gene; 13: *U2AF65B* gene. (C) Melting curves of the reference genes.

The optical density (OD, A260/280 ratio) was higher than 1.8, indicating high purity without contamination. Following agarose gel electrophoresis, the main RNA bands of each sample were clear, without significant degradation, and were used for subsequent experiments (Fig. 1A).

PCR and RT-qPCR were used to confirm the primer specificity. Agarose gel electrophoresis was used to carry out the PCR amplification process, which revealed single sharp product bands of the expected sizes (Fig. 1B). Additionally, RT-qPCR amplification was performed, and each melting curve displayed a clear single peak, indicating the lack of nonspecific products such as primer dimers. These results demonstrate that the primers met the specificity requirements and were suitable for use in RT-qPCR experiments (Fig. 1C).

Plotting a standard curve with the Ct value as the vertical coordinate and the logarithmic value of dilution as the horizontal coordinate allowed us to determine the E and R^2^ values of the putative reference genes (Table 2). Each putative reference genes' R^2^ value varied from 0.9897 to 0.9999, indicating strong correlation with the standard curves. Each primer's amplification efficiency varied from 91.693 to 130.676 %, demonstrating a respectable level of amplification efficiency.

### 3.2 Expression profile analysis of candidate reference genes

**Fig. 2:**
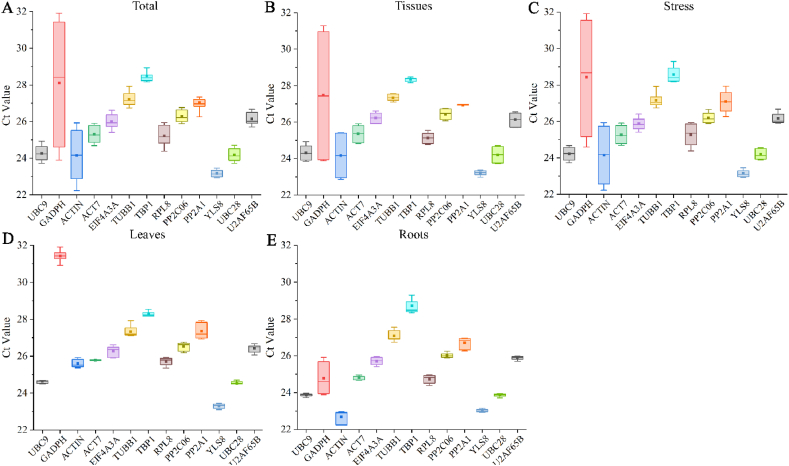
Ct values for 13 Candidate Genes. The boxes illustrate the interquartile range, with the lines in the middle indicating the median. The bottom and top edges of each box represent the 25th and 75th percentiles. The whiskers extending from the boxes approximate the minimum and maximum values, while the small squares within the boxes denote the average values. Results from (A) All samples; (B) The roots and leaves of unstressed seedlings; (C) The roots and leaves of seedlings under different concentrations of Cd stress; (D) Leaves of seedlings under no stress and Cd stress; (E) Roots of seedlings under no stress and Cd stress.

Thirteen putative reference genes' expression levels were evaluated in the samples using RT-qPCR. The Ct value, which indicates the number of cycles needed for the RT-qPCR amplification to meet a predefined threshold, is inversely associated with the levels of gene expression; a lower Ct value indicates higher levels of gene expression. The box plot in Fig. 2 illustrates that the transcript levels of the 13 candidate reference genes were broadly expressed across all tested samples, with Ct values ranging from 22.229 (*ACTIN*) to 31.911 (*GAPDH*). *YLS8* exhibited the least variation in Ct value (23.1591 ± 0.2614), suggesting it had the most stable expression among all samples. Conversely, *GAPDH* showed the greatest variation in Ct value (28.1099 ± 4.0114), proving that it isn't a good reference gene for this research (Fig. 2A–E).

### 3.3 Stability analysis of candidate reference genes

Using three methods (geNorm, NormFinder, BestKeeper) and the online analysis tool RefFinder, we assessed the stability of thirteen putative reference genes of *I. domestica* under Cd stress.

#### 3.3.1 GeNorm analysis

**Fig. 3:**
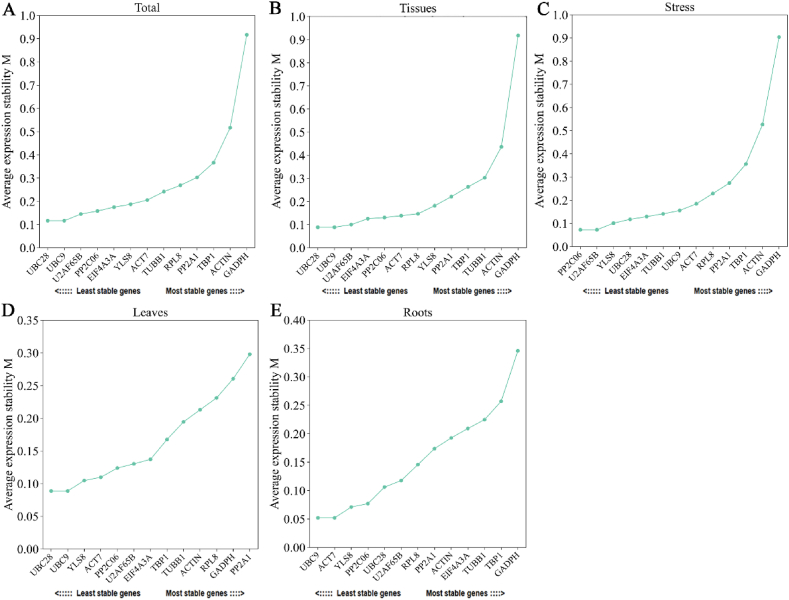
GeNorm was used to analyse the average expression stability (M value) of 13 candidate reference genes. The genes are presented in each chart with stability listed in descending order from left to right, culminating at the bottom. Results from (A) All samples; (B) The roots and leaves of unstressed seedlings; (C) The roots and leaves of seedlings under different concentrations of Cd stress; (D) Leaves of seedlings under no stress and Cd stress; (E) Roots of seedlings under no stress and Cd stress.

GeNorm software was utilized to determine the stability M value of the reference gene by analyzing the Ct value for intergroup variations. Stable expression was defined as an M value below 1.5, with a smaller M value indicating greater stability [30]. The findings revealed that all 13 candidate reference genes exhibited M values less than 1.5 under various experimental conditions, indicating their stable expression (Fig. 3). Moreover, *UBC9* and *UBC28* exhibited the lowest M values in the ‘Total’ sets, leaves and tissues, indicating high stability in most sample combinations (Fig. 3A, B, D). *UBC9* and *ACT7* showed the greatest stability in the root tissues (Fig. 3E). *PP2C06* and *U2AF65B* under cadmium exposure, were the most stable genes (Fig. 3C). In leaf tissue, *PP2A1* was the least stable. Among all other sample combinations, *GAPDH* showed the most unstable stability.

**Fig. 4:**
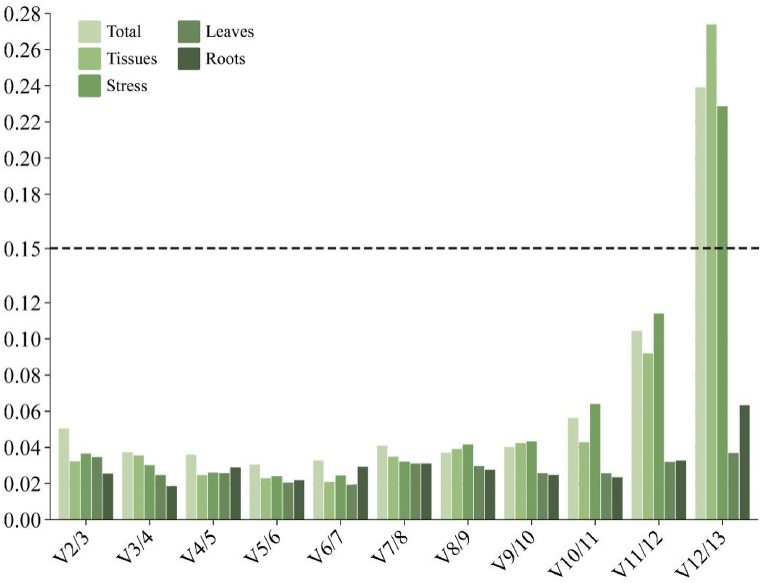
GeNorm Analysis of the Optimal Number (V Value) of 13 Reference Genes. The screening criteria was V/V_n+1_ < 0.15.

In addition, geNorm provides a study of pairwise comparison (Vn/Vn+1) to find the ideal number of reference genes. When Vn/Vn+1 is less than 0.15, this approach shows that n is the ideal number of reference genes. The findings demonstrated that every V2/V3 value was less than 0.15 (Fig. 4), and the other ratios were less than 0.15, except for V_12_/V_13_ values, which were greater than 0.15, indicating that the accuracy of the results could be satisfied by using 2–11 internal reference genes. Two genes were found to be the ideal number of reference genes for all sets, which helped to streamline the experiment and increase the precision of the findings.

In summary, geNorm analysis indicated that *UBC9* and *UBC28* were the most stable reference genes for RT-qPCR normalization in most sample combinations of *I. domestica* under Cd stress, whereas *GADPH* was the least stable. The use of only two reference genes for normalization is recommended.

#### 3.3.2 NormFinder analysis

**Fig. 5:**
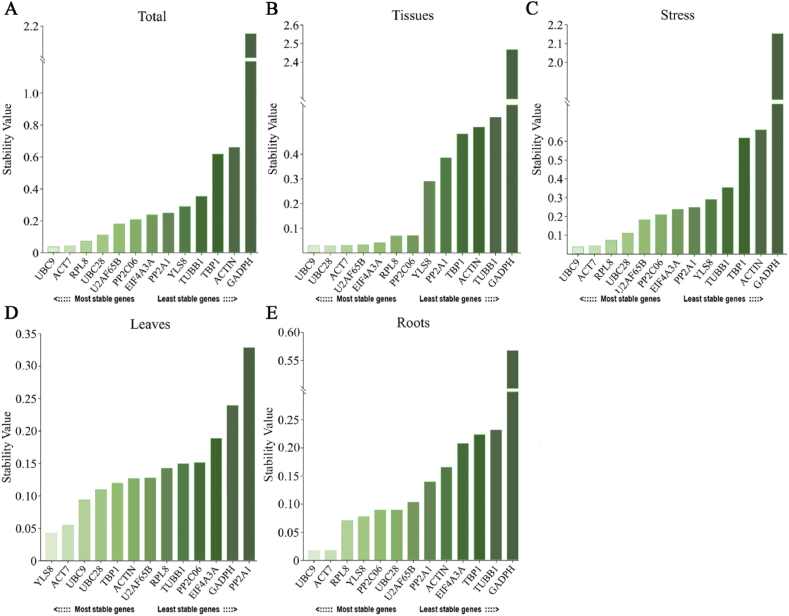
NormFinder analysis of candidate reference genes. Genes are sorted from left to right according to stability. Results from (A) All samples; (B) The roots and leaves of unstressed seedlings; (C) The roots and leaves of seedlings under different concentrations of Cd stress; (D) Leaves of seedlings under no stress and Cd stress; (E) Roots of seedlings under no stress and Cd stress.

NormFinder software is similar to geNorm in that it calculates gene expression stability, but also considers variations within a sample set. SV was calculated by analyzing the variance within and between groups. A smaller SV indicates a greater reference gene stability [31]. According to the findings, *UBC9* was the reference gene that was most stable across all samples (Fig. 5A), different tissues (Fig. 5B), Cd stress (Fig. 5C), and root tissues (Fig. 5E). *YLS8* exhibited the leaf tissues' most consistent expression (Fig. 5D). Among the reference genes that varied most between root tissues and tissues under Cd stress, *GADPH* was the least stable. In the leaves, *PP2A1* exhibited the least stability. The stable reference genes generated by the algorithm were marginally different from those examined by geNorm, but not significantly different. The unstable reference gene results agreed with the geNorm results.

#### 3.3.3 BestKeeper analysis

To evaluate the stability of the reference genes, BestKeeper looked at the coefficient of variation (CV) and standard deviation (SD) of the Ct values. The reference gene was considered to be more stable when the CV and SD were less [32]. As per the BestKeeper analysis shown in Table 3. Of all the samples, *YLS8* remained the most stable under Cd stress. Based on the results, the most stable genes in various tissues were found to be *PP2A1*, *ACT7* in leaves, and *UBC9* in root tissues. *GADPH* was the reference gene that was most unstable in all samples, tissues, Cd stress, and root tissues, while *PP2A1* was the most unstable in leaf tissues. A reference gene was found by the algorithm ranking, which was different from the outcomes of GeNorm and NormFinder by a large margin. Under the majority of experimental situations, *GADPH* continued to be the reference gene with the least stability, which is in line with the findings from geNorm and NormFinder.

**Table 3:** Ranking of the expression stability of 13 candidate reference genes by BestKeeper.

Ranking	Total			Tissues			Stress			Leaves			Roots		
	Genes	SD	CV(%)	Genes	SD	CV(%)	Genes	SD	CV(%)	Genes	SD	CV(%)	Genes	SD	CV(%)
1	*YLS8*	0.15	0.64	*PP2A1*	0.05	0.18	*YLS8*	0.16	0.68	*ACT7*	0.08	0.31	*UBC9*	0.06	0.27
2	*TUBB1*	0.26	0.95	*TBP1*	0.10	0.34	*PP2C06*	0.24	0.92	*UBC9*	0.08	0.34	*YLS8*	0.07	0.29
3	*PP2C06*	0.27	1.03	*YLS8*	0.13	0.54	*U2AF65B*	0.24	0.92	*UBC28*	0.10	0.42	*UBC28*	0.07	0.30
4	*TBP1*	0.27	0.95	*TUBB1*	0.15	0.54	*UBC28*	0.26	1.09	*YLS8*	0.11	0.45	*U2AF65B*	0.09	0.33
5	*U2AF65B*	0.29	1.10	*PP2C06*	0.29	1.10	*TUBB1*	0.27	0.99	*TBP1*	0.11	0.39	*ACT7*	0.09	0.35
6	*EIF4A3A*	0.30	1.16	*EIF4A3A*	0.30	1.13	*EIF4A3A*	0.27	1.05	*RPL8*	0.19	0.73	*PP2C06*	0.10	0.40
7	*UBC28*	0.33	1.35	*RPL8*	0.30	1.18	*TBP1*	0.35	1.22	*U2AF65B*	0.19	0.72	*EIF4A3A*	0.16	0.63
8	*PP2A1*	0.35	1.31	*U2AF65B*	0.39	1.47	*UBC9*	0.36	1.49	*ACTIN*	0.20	0.76	*RPL8*	0.20	0.82
9	*UBC9*	0.38	1.58	*UBC9*	0.43	1.76	*PP2A1*	0.48	1.77	*PP2C06*	0.20	0.76	*PP2A1*	0.27	1.01
10	*ACT7*	0.48	1.89	*UBC28*	0.45	1.87	*ACT7*	0.48	1.90	*TUBB1*	0.23	0.83	*TUBB1*	0.27	1.00
11	*RPL8*	0.49	1.93	*ACT7*	0.48	1.88	*RPL8*	0.58	2.31	*EIF4A3A*	0.23	0.88	*ACTIN*	0.30	1.33
12	*ACTIN*	1.46	6.04	*ACTIN*	1.24	5.13	*ACTIN*	1.57	6.50	*GADPH*	0.26	0.83	*TBP1*	0.34	1.18
13	*GADPH*	3.32	11.82	*GADPH*	3.57	12.97	*GADPH*	3.20	11.27	*PP2A1*	0.35	1.27	*GADPH*	0.69	2.78

#### 3.3.4 RefFinder analysis

**Fig. 6:**
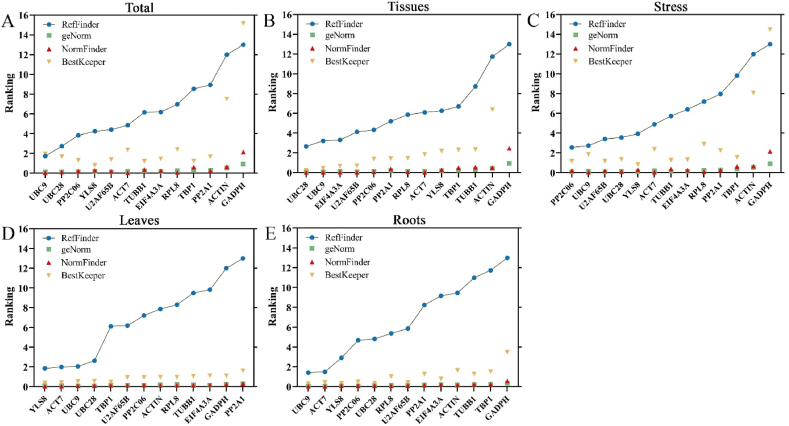
Comprehensive ranking of 13 candidate reference genes. Results from (A) All samples; (B) The roots and leaves of unstressed seedlings; (C) The roots and leaves of seedlings under different concentrations of Cd stress; (D) Leaves of seedlings under no stress and Cd stress; (E) Roots of seedlings under no stress and Cd stress.

Three software packages (geNorm, NormFinder, and BestKeeper) were used to calculate the rankings of the best reference genes. The results showed considerable differences between the ranks. Consequently, we computed the geometric mean of the ranking outcomes of the three software programs using RefFinder (https://blooge.cn/RefFinder/), an online analysis tool, to get an overall rating of the reference genes [33]. Fig. 6 presents the results of the comprehensive analysis using RefFinder. *UBC9* and *UBC28* were consistently ranked as the top two reference genes across all samples and tissues (Fig. 6A; B), in agreement with the geNorm calculation results, and similar to the NormFinder results. Therefore, *UBC9* and *UBC28* may be considered the most stable reference genes. In the other sample combinations, the top two overall rankings in RefFinder were consistent with the top four or five positions in other software. It was found that *UBC9* and *PP2C06* were the most stable in leaf tissues during Cd stress (Fig. 6D). In the root tissues, *UBC9* and *ACT7* were identified as the most stable (Fig. 6E). In Cd stress-related studies, *GADPH* was determined to be an unsuitable reference gene for *I. domestica* due to its lack of stability compared to other sample combinations (Fig. 6C).

**Fig. 7:**
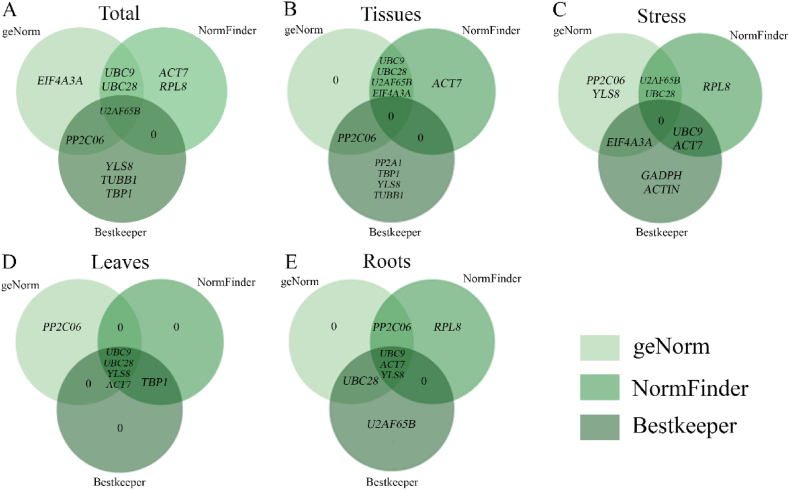
The Venn diagram shows the overlap between the top five stable reference genes identified by geNorm, NormFinder and BestKeeper. The overlapping genes were confirmed by multiple algorithms and had high confidence. The three green colours, from light to dark, represent the geNorm, NormFinder and BestKeeper analyses. Results from (A) All samples; (B) The roots and leaves of unstressed seedlings; (C) The roots and leaves of seedlings under different concentrations of Cd stress; (D) Leaves of seedlings under no stress and Cd stress; (E) Roots of seedlings under no stress and Cd stress. (For interpretation of the references to colour in this figure legend, the reader is referred to the Web version of this article.)

Lastly, Venn diagrams were used to display the five reference genes that were found to be the most stable using the geNorm, NormFinder, and BestKeeper algorithms (Fig. 7A–E). The two most stable reference genes for each sample combination were compatible with the computed results from at least one of the three software systems, according to a thorough RefFinder investigatio. This indicates that the results of the RefFinder comprehensive ranking can be used as a reliable reference for screening the best reference genes.

### 3.4 Validation of selected reference genes

**Fig. 8:**
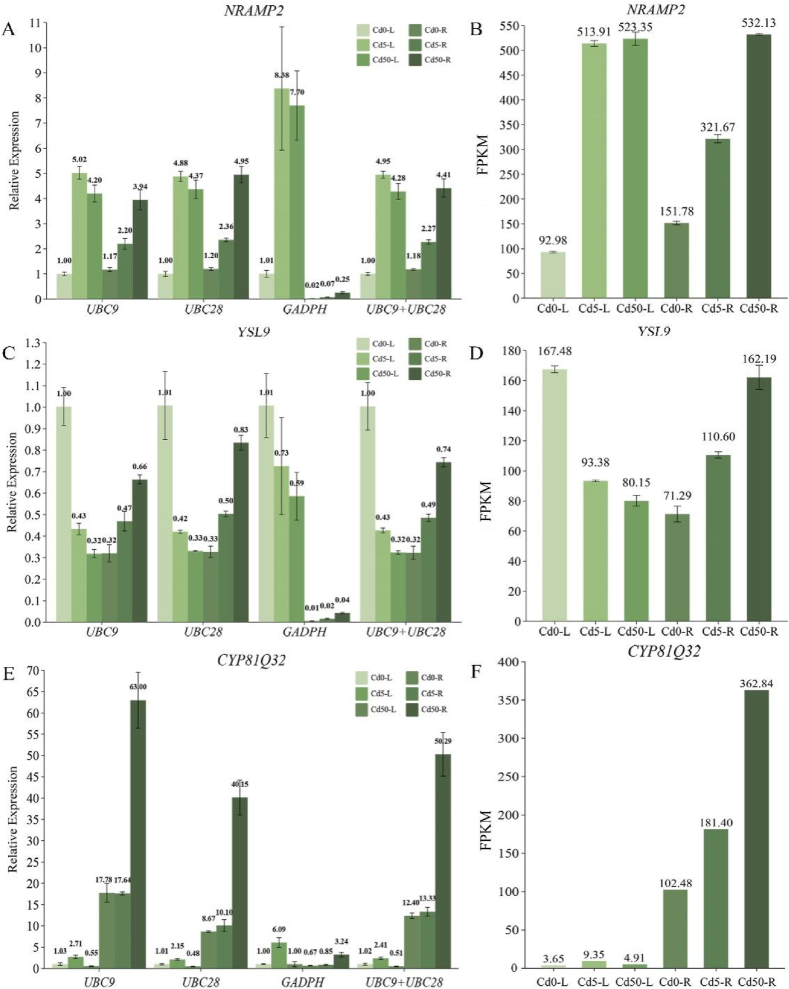
The accuracy of the results of the selected reference genes was verified using the individually or combination stable reference genes and transcriptome sequencing. (A) relative expression of the *NRAMP2* gene; (B) transcriptome sequencing results of the *NRAMP2* gene; (C) relative expression of the *YSL9* gene; (D) transcriptome sequencing results of the *YSL9* gene; (E) relative expression of the *CYP81Q32* gene; (F) transcriptome sequencing results of the *CYP81Q32* gene.

*NRAMP2*, *YSL9*, and *CYP81Q32* transcriptomic differential genes were utilized to confirm the accuracy of the chosen reference genes. The expression levels were determined by applying the 2^-ΔΔCt^ technique to compute the Ct values. To evaluate the validity of the chosen reference genes, RT-qPCR assays were conducted using the two most stable reference genes, either separately or in conjunction with the least stable reference gene found by the RefFinder comprehensive analysis. The results indicated that when the most stable reference genes, *UBC9*, *UBC28*, and the combination *UBC9*+*UBC28*, were used, the expression trends and levels of the three target genes were consistent, and the Ct values for each sample were similar across the different stable reference genes and combinations. Despite the fact that the expression pattern for *GADPH*, the most unstable reference gene, was in line with that of the other reference genes, the expression levels in the roots were significantly different (Fig. 8A; C; E). Specifically, when *UBC9*, *UBC28*, and *UBC9*+*UBC28* were used as reference genes, the CT value of *NRAMP2* was 1.17–4.95, while when *GADPH* was used as a reference gene, the CT value of *NRAMP2* was only 0.02–0.25. Similarly, when *GADPH* was used as the internal reference gene for *YSL9* and *CYP81Q32*, the Ct values of the three root samples were significantly lower (Fig. 8A; C; E).

The FPKM values of the three target genes in the transcriptome sequencing results were used as references (Fig. 8B; D; F). The relative expression and trend of *UBC9*, *UBC28*, and *UBC9*+*UBC28* when they were used as reference genes were essentially the same as the expression and trend of *NRAMP2*, *YSL9*, and *CYP81Q32* in the transcriptome sequencing results. When *GADPH* was utilized as the reference gene, there was a notable variation in its relative expression, though, and this was seen in the expression of the gene in the roots (Fig. 8A; C; E). Reference genes are not fixed in research settings, and proper gene expression normalization depends on the selection of relevant reference genes for particular research contexts.

In summary, *UBC9* and *UBC28* exhibited strong and stable expression at root and leaf sites in *I. domestica* under Cd stress, making them appropriate reference genes for this study.

## 4 Discussion

Gene expression analysis offers significant benefits to the investigation of molecular mechanisms. RT-qPCR is a widely used molecular biology technique for measuring the expression level of genes. The precision and dependability of the data produced by this method are significantly influenced by the stability of the reference gene used in the process [17,18]. In the test samples, the reference genes should ideally be similarly expressed. However, gene expression at the transcriptional level in tissues is dynamic and no absolute reference gene has been identified. Gene expression tends to fluctuate according to species, site, developmental stage, and environment [20,24,40]. Some uncontrolled environmental factors in the study, for example, under the same Cd-treated, the light, water, temperature of the environment or the genotype of the research material and the seedling age will cause changes in the reference gene and the target gene's expression [29,37,41]. As a result, it's crucial to choose relevant reference genes for a variety of experimental setups and confirm that genes express themselves steadily when combined with particular environmental elements.

GeNorm, NormFinder, BestKeeper, and RefFinder were used to evaluated the stability of reference genes [[30], [31], [32], [33]]. We found that the three programs ranked the candidate reference genes differently and that the ranks of BestKeeper differed considerably from those of both geNorm and NormFinder. This difference was also observed in *I. germanica* L., *Chrysanthemum* spp., and *L. megaphylla* [24,27,42]. The results of NormFinder have been reported to differ from those of RefFinder, not only because of the efficiency values, but also because the original software can calculate inter- and intragroup variance, but RefFinder does not allow the user to form groups [40]. In this investigation, we employed the NormFinder analysis ranking outcomes from the first software, and the ranking results from RefFinder for comprehensive ranking. The roots and leaves of the *I. domestica* seedlings under Cd stress constituted the entire sample used in this study. We again examined various sample types in detail when calculating using each software, and discovered that they had different stable reference genes. Using RefFinder as an example, *UBC9* and *UBC28* were the best among all samples, whereas *UBC28* ranked 4th or 5th under both stress conditions and in leaves or roots. In contrast, the activity of *PP2C06* was the highest under both stress conditions and in leaf tissues, which may be related to the mechanism of action of the *PP2C* family [43]. This demonstrated that the stable reference genes for all samples were *UBC9* and *UBC28*, with or without Cd stress, whereas *PP2C06* was more accurate when leaf and root tissues or Cd stress were analyzed separately. However, some reference gene studies have been conducted, such as those examining the optimal reference gene for all samples under specific stress or comparing various stresses. For example, in Cd-, Pb-, and salt-stressed roots, *EIF-5A* and *UBC* were the best reference genes for *I. lactea* var. *chinensis* [29]. *EIF3* and *ACT* were the best reference genes for *Broussonetia papyrifera* under drought, salt, and ZnSO_4_ stress, whereas heat shock proteins and NADH dehydrogenase genes were the most suitable under heavy metal stress [44]. However, for a detailed study of Cd stress, samples from a particular tissue or treatment should be studied individually, and a detailed sample analysis can provide a more accurate reference. Reference gene studies with similar detailed groupings have been reported for species such as *Avena sativa* L., *L. megaphylla*, *I. lactea* var. *chinensis*, and garlic [24,[45], [46], [47]]. In general, *UBC9* and *UBC28* were among the four most abundant sample types, indicating that they were the most effective reference genes for *I. domestica* under Cd stress.

*GADPH* and *ACT* have been used to study molecular biology drugs in *I. domestica* [3,16]. Reference genes of *I. domestica* under drought stress conditions were screened. The best reference genes in the rhizome under drought stress were *ACT* and *EF1β*, whereas *EF1β* and *UBC9* were the best in unstressed plants [28]. In addition to reflecting current gene expression in tissues, transcriptome sequencing has recently been used to identify reference genes [21,23,25]. Our study showed that the *UBC9* and *UBC28* reference genes identified by transcriptome analysis were the most stable, whereas the *GADPH* and *ACT* reference genes were unstable. *UBC9* and *ACT11* were the best reference genes for various tissues and developmental stages in *I. germanica* L. hybrid self-selected variants [26]. In three kinds of *I. germanica* L., at different stages of flower development, the most stable reference genes were *IgUBC* and *IgGAPDH* [27]. The reference genes' levels of expression, TIP41-like family protein (*TIP41*), cyclophilin (*CYP*), cytosolic phosphoglycerate kinase (*PGK*), *GAP*, and *PP2A*s were relatively stable in the leaves of *I. lactea* var. *chinensis* under low-temperature, salt, PEG, Cu, and Pb stress [46], whereas the expression of *EIF-5A* and *UBC* was most stable in the roots of this species under Pb, Cd, and salt stress [29]. Comparison and analysis of the results of this study with stable reference genes of closely related species revealed that members of the *UBC* gene family are stably expressed under different conditions in *Iris* species, and these genes can be considered as common reference genes. This study included *UBC9* and *UBC28*. Other common reference genes, *GADPH* and *ACT*, showed differences depending on the experimental conditions. It should be noted that some of the reference genes reported in *I. domestica* were not stably expressed as homologous genes in our transcriptomes; only 13 reference genes were analyzed in this study. Transcriptome sequencing may not have produced precise annotations because of the lack of genomic information available for this species.

Cd is a highly toxic heavy metal for plants. Researchers have investigated the molecular mechanisms that reduce Cd uptake in medicinal plants and the environmental remediation through Cd uptake by hyperaccumulator plants [29,35,48]. For roots stressed with Hg, Pb, As, and Cr, and for leaves treated with Cd, Hg, Cr, and As, *U2AF* was the most stable reference gene in *Panicum virgatum* L. The best reference gene was found to be *18S rRNA*, whereas *CYP5* was better for root tissues that had been exposed to Cd [37]. In *Salix viminalis*, under Cd, Ni, Zn, and Cu stress, *TIP41* and cyclin-dependent kinase (*CDC2*) were the most stable genes, whereas *GAPDH* was the least stable. Furthermore, despite conditions of heat, cold, salt, and dehydration, *CDC2* expression remained steady [41]. *GAPDH* was the least stable gene in *I. domestica* under Cd stress, whereas *UBC9* and *UBC28* were the most stable. This differs from other species under Cd or other heavy metal stress. Suitable reference genes vary between species and experimental conditions, and their selection should be specific. Even within the same species, stable reference genes can differ across developmental stages, environments, or experimental conditions. These results emphasize how crucial it is to choose the right reference genes for various experimental setups. Therefore, when using reference genes under varying environmental conditions or in different developmental stage, we should first refer to the reported reference genes of near-source species to determine the range of reference genes. Secondly, with the popularization of transcriptome sequencing applications, when the study has transcriptome data, it should be selected for further use with stable expression in transcriptome data. When there is no transcriptome as a reference, candidate reference genes should be extensively screened to obtain suitable reference genes for specific applications.

We selected two differentially expressed genes related to metal ion transport, *NRAMP2*, *YSL9*, and *CYP81Q32*, which are related to the medicinal ingredient isoflavone, to verify the selected internal reference genes. To examine the relative expression levels of these genes, we employed the most unstable reference gene, *GADPH*, and the best reference genes, *UBC9*, *UBC28*, and *UBC9+UBC28*. Accuracy was evaluated in conjunction with the gene expression in the transcriptome. The outcomes demonstrated that the expression trend of stable reference genes was similar to that of the transcriptome, whereas the expression of unstable reference genes varied greatly. In contrast, the quantification of *NRAMP2* revealed similar expression levels in Cd5-L, Cd50-L, and Cd50-R. The transcriptome analysis demonstrated a gradual increase in these genes' expression, with a slight decrease observed following the use of *UBC9.* When *UBC28* was used, the highest expression in Cd50-R agreed with that of the transcriptome, whereas of the expression in Cd50-L was slightly lower than that of Cd5-L. This phenomenon may have been caused by a small error in the gene expression in the transcriptome. For samples with very similar expression, the relative quantitative results of the target gene fluctuated slightly, owing to the instability of the reference gene. For quantitative results to be accurate during reference gene validation, comparison with the transcriptomes' genes' FPKM is crucial [23,49,50]. Therefore, stable reference genes and target genes with significant differences in expression should be chosen as much as possible for RT-qPCR, and the differences in expression between the two genes should not be excessive.

## 5 Conclusions

In this study, we investigated the reference genes *UBC9* and *UBC28* in the roots and leaves of *I. domestica* seedlings exposed to Cd stress for the first time. These reference genes were used to evaluate the expression of the metal ion response genes *NRAMP2*, *YSL9* and *CYP81Q32* in *I. domestica*, and both the reference genes' appropriateness and the transporter genes' expression patterns were validated. These findings serve as a reference for investigating gene expression in *I. domestica* and its relatives under Cd stress, as well as laying the groundwork for future molecular biology research in this plant.

## Funding

This work was supported by S&T Program of Hebei (Grant No. 21326317D).

## Data availability statement

The transcriptome profiling data used in this study have been deposited in the China National Center for Bioinformation (CNCB) database under the accession number CRA018277.

## CRediT authorship contribution statement

**Beibei Su:** Writing – review & editing, Data curation, Conceptualization. **Ziwei Li:** Writing – original draft, Software, Data curation. **Hongli Liu:** Validation, Methodology. **Xiaoyun Niu:** Supervision. **Xiaojie Zhao:** Validation. **Yumeng Wu:** Investigation. **Qian Wang:** Visualization. **Yangchen Yuan:** Resources. **Zhuolin Xiao:** Formal analysis. **Dazhuang Huang:** Project administration, Funding acquisition.

## Declaration of competing interest

The authors declare that they have no known competing financial interests or personal relationships that could have appeared to influence the work reported in this paper.

## References

[1] Xu W., Yu F., Jia Q., Luo G., Bi X. (2017). ‘Sweet princess’: a new summer ornamental Iris cultivar. Hortscience.

[2] Xu W., Luo G., Yu F., Jia Q., Zheng Y., Bi X., Lei J. (2018). Characterization of anthocyanins in the hybrid progenies derived from *Iris dichotoma* and *I. domestica* by HPLC-DAD-ESI/MS analysis. Phytochemistry.

[3] Tian M., Zhang X., Zhu Y., Xie G., Qin M. (2018). Global transcriptome analyses reveal differentially expressed genes of six organs and putative genes involved in (Iso)flavonoid biosynthesis in *Belamcanda chinensis*. Front. Plant Sci..

[4] Woźniak D., Matkowski A. (2015). *Belamcandae chinensis rhizoma* – a review of phytochemistry and bioactivity. Fitoterapia.

[5] Chen Y.J., Liang Z.T., Zhu Y., Xie G.Y., Tian M., Zhao Z.Z., Qin M.J. (2014). Tissue-specific metabolites profiling and quantitative analyses of flavonoids in the rhizome of Belamcanda chinensis by combining laser-microdissection with UHPLC-Q/TOF-MS and UHPLC–QqQ-MS. Talanta.

[6] Ding L., Liu R., Gao Y., Xiao J., Lv Y., Zhou J., Zhang Q. (2023). Effect of tetraploidization on morphological and fertility characteristics in *Iris × norrisii* Lenz. Sci. Hortic..

[7] Gao J., Cai W., Xiao Y., Yu F., Zheng Y., Bi X. (2021). Variation and inheritance of the degree of style branching in hybrids of *Iris dichotoma × I. domestica*. Sci. Hortic..

[8] Wang P., Chen H., Kopittke P.M., Zhao F.-J. (2019). Cadmium contamination in agricultural soils of China and the impact on food safety. Environmental Pollution.

[9] Tang J., Liu Q., Yuan H., Zhang Y., Huang S. (2018). Molecular analysis of a novel alkaline metal salt (NaCl)-responsive WRKY transcription factor gene *IlWRKY1* from the halophyte *Iris lactea* var. *chinensis*. Int. Biodeterior. Biodegrad..

[10] Liu Q., Zhang Y., Wang Y., Wang W., Gu C., Huang S., Yuan H., Dhankher O.P. (2020). Quantitative proteomic analysis reveals complex regulatory and metabolic response of *Iris lactea* Pall. var. *chinensis* to cadmium toxicity. J. Hazard Mater..

[11] Sheng L., Zhao W., Yang X., Mao H., Zhu S. (2023). Response characteristics of rhizosphere microbial community and metabolites of *Iris tectorum* to Cr stress. ECOTOX ENVIRON SAFE.

[12] Zhang J., Huang D., Zhao X., Zhang M., Wang Q., Hou X., Di D., Su B., Wang S., Sun P. (2022). Drought-responsive WRKY transcription factor genes *IgWRKY50* and *IgWRKY32* from *Iris germanica* enhance drought resistance in transgenic *Arabidopsis*. Front. Plant Sci..

[13] Zhao Z., Li T., Cheng Y., Wang F., Zhao X. (2021). Morphological and metabolic responses of four *Iris germanica* cultivars under salinity stress. Sci. Hortic..

[14] Iwashina T., Mizuno T. (2020). Flavonoids and xanthones from the genus *Iris*: phytochemistry, relationships with flower colors and taxonomy, and activities and function. Nat. Prod. Commun..

[15] Zhu Y., Pu B., Xie G., Tian M., Xu F., Qin M. (2014). Dynamic changes of flavonoids contents in the different parts of rhizome of *Belamcanda chinensis* during the thermal drying process. Molecules.

[16] Zhang X., Zhu Y., Ye J., Ye Z., Zhu R., Xie G., Zhao Y., Qin M. (2021). *Iris domestica* (Iso)flavone 7- and 3′-O-glycosyltransferases can Be induced by CuCl2. Front. Plant Sci..

[17] Kou X., Zhang L., Yang S., Li G., Ye J. (2017). Selection and validation of reference genes for quantitative RT-PCR analysis in peach fruit under different experimental conditions. Sci. Hortic..

[18] Bustin S.A., Vladimir B., Garson J.A., Jan H., Jim H., Mikael K., Reinhold M., Tania N., Pfaffl M.W., Shipley G.L. (2009). The MIQE guidelines: minimum information for publication of quantitative real-time PCR experiments. Clin. Chem..

[19] Fleige S., Pfaffl M.W. (2006). RNA integrity and the effect on the real-time qRT-PCR performance. Mol. Aspects Med..

[20] Song H., Mao W., Duan Z., Que Q., Zhou W., Chen X., Li P. (2020). Selection and validation of reference genes for measuring gene expression in *Toona ciliata* under different experimental conditions by quantitative real-time PCR analysis. BMC Plant Biol..

[21] Czechowski T., Stitt M., Altmann T., Udvardi M.K., Scheible W.-R. (2005). Genome-wide identification and testing of superior reference genes for transcript normalization in arabidopsis. Plant Physiol.

[22] Umadevi P., Suraby E.J., Anandaraj M., Nepolean T. (2019). Identification of stable reference gene for transcript normalization in black pepper-*Phytophthora capsici* pathosystem. Physiol. Mol. Biol. Plants.

[23] Yi S., Lin Q., Zhang X., Wang J., Miao Y., Tan N. (2020). Selection and validation of appropriate reference genes for quantitative RT-PCR analysis in *Rubia yunnanensis* diels based on transcriptome data. BioMed Res. Int..

[24] Liu H., Liu J., Chen P., Zhang X., Wang K., Lu J., Li Y. (2023). Selection and validation of optimal RT-qPCR reference genes for the normalization of gene expression under different experimental conditions in *Lindera megaphylla*. Plants.

[25] Yao Y., Wang X., Chen B., Zheng S., Wang-Pruski G., Chen X., Guo R. (2023). Evaluation of reference genes suitable for gene expression during root enlargement in cherry radish based on transcriptomic data. Horticulturae.

[26] Wang Y.J., Zhang Y., Liu Q., Liu L., Huang S., Yuan H. (2021). Reference gene selection for qRT-PCR normalization in *Iris germanica* L. Phyton.

[27] Wang Y.J., Zhang Y.X., Liu Q.Q., Tong H.Y., Zhang T., Gu C.S., Liu L.Q., Huang S.Z., Yuan H.Y. (2021). Selection and validation of appropriate reference genes for RT-qPCR analysis of flowering stages and different genotypes of *Iris germanica* L. Sci. Rep..

[28] Ai Q., Liu C., Han M., Yang L. (2022). Selection and verification of reference genes for qRT-PCR analysis in *Iris domestica* under drought. PHYTON-INT J EXP BOT.

[29] Gu C., Liu L., Xu C., Zhao Y., Zhu X., Huang S. (2014). Reference gene selection for quantitative real-time RT-PCR normalization in *Iris. Lactea* var. *chinensis* roots under cadmium, lead, and salt stress conditions. Sci. World J..

[30] Vandesompele J., Preter K.D., Pattyn F., Poppe B., Roy N.V., Paepe A.D., Speleman F. (2002). Accurate normalization of real-time quantitative RT-PCR data by geometric averaging of multiple internal control genes. Genome Biol..

[31] Andersen C.L., Jensen J.L., Ørntoft T.F. (2004). Normalization of real-time quantitative reverse transcription-PCR data: a model-based variance estimation approach to identify genes suited for normalization, applied to bladder and colon cancer data sets. Cancer Res..

[32] Pfaffl M.W., Tichopad A., Prgomet C., Neuvians T.P. (2004). Determination of stable housekeeping genes, differentially regulated target genes and sample integrity: BestKeeper-Excel-Based tool using pair-wise correlations, Biotechnol. Lett.

[33] Xie F., Wang J., Zhang B. (2023). RefFinder: a web-based tool for comprehensively analyzing and identifying reference genes. Funct. Integr. Genomics.

[34] Fan P., Wu L., Wang Q., Wang Y., Luo H., Song J., Yang M., Yao H., Chen S. (2023). Physiological and molecular mechanisms of medicinal plants in response to cadmium stress: current status and future perspective. J. Hazard Mater..

[35] Rasheed A., Al-Huqail A.A., Ali B., Alghanem S.M.S., Shah A.A., Azeem F., Rizwan M., Al-Qthanin R.N., Soudy F.A. (2024). Molecular characterization of genes involved in tolerance of cadmium in *Triticum aestivum* (L.) under Cd stress. J. Hazard Mater..

[36] Li W., Li J., Hussain K., Peng K., Yu J., Xu M., Yang S. (2024). Transporters and phytohormones analysis reveals differential regulation of ryegrass (*Lolium perenne* L.) in response to cadmium and arsenic stresses. J. Hazard Mater..

[37] Zhao J., Zhou M., Meng Y. (2020). Identification and validation of reference genes for RT-qPCR analysis in switchgrass under heavy metal stresses. Genes.

[38] Li G., Xu G., Lin Z., Li H., Liu Z., Xu Y., Zhang H., Ji R., Luo W., Qiu Y. (2021). Selection of suitable reference genes for RT-qPCR normalisation in sweet potato (Ipomoea batatas L.) under different stresses. J HORTIC SCI BIOTECH.

[39] Bardou P., Mariette J., Escudié F., Djemiel C., Klopp C. (2014). jvenn: an interactive Venn diagram viewer. BMC Bioinf..

[40] Vera Hernández F.P., Martínez Núñez M., Ruiz Rivas M., Vázquez Portillo R.E., Bibbins Martínez M.D., Luna Suárez S., Rosas Cárdenas F.d.F., Flemetakis E. (2018). Reference genes for RT‐qPCR normalisation in different tissues, developmental stages and stress conditions of amaranth. Plant Biol..

[41] Ambroise V., Legay S., Guerriero G., Hausman J.-F., Cuypers A., Sergeant K. (2019). Selection of appropriate reference genes for gene expression analysis under abiotic stresses in *Salix viminalis*. Int. J. Mol. Sci..

[42] Qi S., Yang L., Wen X., Hong Y., Song X., Zhang M., Dai S. (2016). Reference gene selection for RT-qPCR analysis of flower development in *Chrysanthemum morifolium* and *Chrysanthemum lavandulifolium*. Front. Plant Sci..

[43] Bhaskara G.B., Wong M.M., Verslues P.E. (2019). The flip side of phospho-signalling: regulation of protein dephosphorylation and the protein phosphatase 2Cs. Plant Cell Environ..

[44] Chen M., Wang Z., Hao Z., Li H., Feng Q., Yang X., Han X., Zhao X. (2023). Screening and validation of appropriate reference genes for real-time quantitative PCR under PEG, NaCl and ZnSO4 treatments in *Broussonetia papyrifera*. Int. J. Mol. Sci..

[45] Yin H., Yin D., Zhang M., Gao Z., Tuluhong M., Li X., Li J., Li B., Cui G. (2022). Validation of appropriate reference genes for qRT–PCR normalization in oat (*Avena sativa* L.) under UV-B and high-light stresses. Int. J. Mol. Sci..

[46] Gu C., Liu L., Deng Y., Zhu X., Lu X., Huang S. (2014). Validation of reference genes for RT-qPCR normalization in *Iris. lactea* var. *chinensis* leaves under different experimental conditions. Sci. Hortic..

[47] Wang G., Tian C., Wang Y., Wan F., Hu L., Xiong A., Tian J. (2019). Selection of reliable reference genes for quantitative RT-PCR in garlic under salt stress. PeerJ.

[48] Liu Z., Gu C., Chen F., Yang D., Wu K., Chen S., Jiang J., Zhang Z. (2012). Heterologous expression of a Nelumbo nucifera phytochelatin synthase gene enhances cadmium tolerance in *Arabidopsis thaliana*. Appl. Biochem. Biotechnol..

[49] Bai X., Chen T., Wu Y., Tang M., Xu Z.-F. (2021). Selection and validation of reference genes for qRT-PCR analysis in the oil-rich tuber crop tiger nut (*Cyperus esculentus*) based on transcriptome data. Int. J. Mol. Sci..

[50] Zheng Y., Ma Y., Luo J., Li J., Zheng X., Gong H., Deng L., Zhao G., Luo C., Liu X., Wu H. (2023). Identification and analysis of reference and tissue-specific genes in bitter gourd based on transcriptome data. Horticulturae.

